# Design of Laves phase-reinforced compositionally complex alloy

**DOI:** 10.1038/s41598-023-43722-6

**Published:** 2023-10-06

**Authors:** Gerald Ressel, Florian Biermair, Simon Fellner, Christoph Gammer, Vsevolod I. Razumovskiy

**Affiliations:** 1 Materials Center Leoben Forschung GmbH, Roseggerstraße 12, 8700 Leoben, Austria; 2 Erich Schmid Institute of Materials Science, Austrian Academy of Sciences, Jahnstrasse 12, 8700 Leoben, Austria

**Keywords:** Structural materials, Mechanical properties, Metals and alloys

## Abstract

Topologically close-packed (TCP) phases such as Laves phases are usually considered to harm the mechanical properties of classical superalloys for high-temperature applications. However, if an optimal fraction and size are designed, this situation can completely change for some compositionally complex alloys (CCA). Based on existing studies on austenitic or ferritic steels, we propose in this paper a design strategy aimed at exploiting the role of the Laves phase in defining the mechanical properties of wrought CCAs at elevated temperatures. We demonstrate its efficiency by applying it to the design and production of a new Laves phase—reinforced CCA and present the results of their experimental and theoretical investigation. The results show that a new Laves phase-reinforced CCA can have fine-grained microstructures, lower density, and superior mechanical strength at elevated temperatures while maintaining workability. These new alloys show promising properties compared to existing CCA wrought alloys and actual Ni-based superalloys.

## Introduction

Innovative and highly efficient energy conversion and propulsion systems driven by so-called green hydrogen, bio-, and synthetic fuels have become highly relevant in multiple energy-producing and transportation industries. However, constructing such efficient systems is limited to using standard materials, such as Ni-based superalloys. Compositionally complex alloys (CCA), comprising 4 to 5 base elements with concentrations between 5 and 35 at.%, have already been proposed as a promising alternative to Ni-base and other classical alloys typically based on one principal element^1–3^. CCAs provide access to a wide range of chemical compositions, and even slight chemical changes can have pronounced effects on material microstructure and properties. Thus, it is no surprise that this relatively new class of alloys has already attracted much attention in both industry and academia. A direct application of these alloys in industrial applications, however, requires several significant development steps on the way to achieving desired material properties, such as a unique combination of specific strength, ductility^4,5^, oxidation resistance^6,7^, all at ambient and high temperatures, and control of grain sizes and workability. All these aspects would make CCAs a perfect candidate for aerospace- and energy production industries that constantly need new materials and material design concepts aiming at better performance in combination with new material development strategies.

At the current stage, the CCA development is based on the original concept of high entropy alloys (HEAs), which focuses on the stabilization of a single-phase solid solution that is believed to provide the ultimate material performance^1,8^. The related concept of CCAs extends the concept of HEAs to minor deviations of nearly equivalent concentrations of principal elements and minor alloying additions. This concept leads to the formation of a multi-phase microstructure with coherent precipitates^3^, similar to a concept used in precipitation-hardened Ni-base superalloys^9^. The formation of a multi-phase microstructure has been shown to be specifically important for high-temperature applications, where the formation of a microstructure with nano-scaled, coherent L1$_2$ particles yields a significant gain in strength properties and allows the material to withstand high static and cyclic mechanical loads at temperatures beyond 600 °C^9^.

A few recent CCA design concepts include the formation of a two-phase microstructure enhanced with coherent geometrically closed packed (GCP) phases (typically $\gamma^{\prime}$ or $\gamma^{\prime\prime}$) to ensure alloy performance at elevated temperatures^3^, including improved creep properties^10,11^. The topologically close-packed (TCP) phases on the other hand, such as $\sigma$ or Laves, are traditionally treated as “undesirable” in most of the alloys^1,8^, especially in plate-like form, as they are generally brittle and lead to deterioration of mechanical properties^12,13^. The phase stability of most of the designed state-of-the-art CCAs is usually carefully checked to avoid precipitation of those phases^14–16^, whereas others^17^ already proposed the strengthening effect of $\sigma$ or $\mu$ phases. In literature, there have been some indications that small amounts of Laves phases can be beneficial under certain conditions in classical alloys, such as ferritic or austenitic high-temperature steels^18–25^, which, however, has never been proven to be valid for other alloys such as CCAs.

Based on these studies, we would like to present a CCA design concept focusing on the positive effect of an intended GCP and Laves phase precipitation on the specific strength, grain size control, and workability of wrought precipitation strengthened FeCoCrNi-base alloys. The alloy design concept is based on density functional theory (DFT) and Calphad-based modeling of the desired lattice misfit between the matrix and the GCP precipitates, as well as appropriate chemical composition and heat treatment for precipitation of the desired amounts of GCP and Laves phases. After the computational design, the novel alloys are produced by casting and thermo-mechanical processing. They are finally studied regarding adjusted grain sizes and phase fractions, as well as their chemical compositions using a combination of state-of-the-art methods, such as scanning-, transmission electron microscopy (SEM, TEM), and atom probe tomography (APT) methods. Furthermore, the strengths of the materials and their deformation mechanisms have been determined by means of high-temperature compression tests and subsequent bright-field scanning TEM (BF-STEM) investigations.

## Results

### Alloy design concept

**Figure 1:**
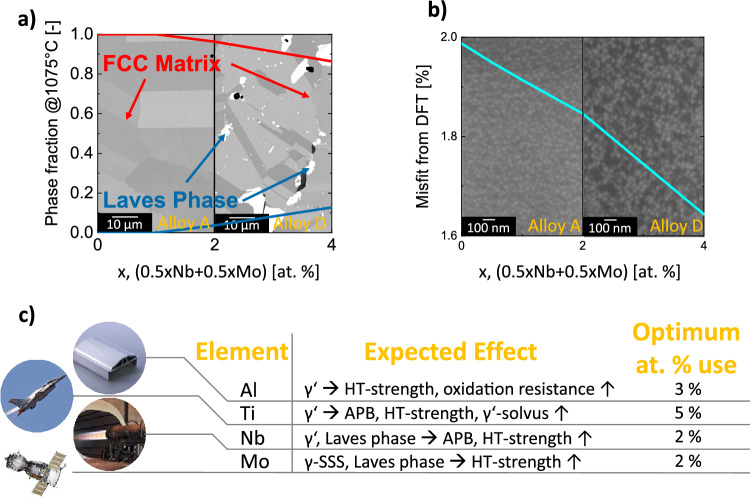
(**a**) Calphad calculations of the fcc matrix (red line) and Laves phase (blue line) fractions at the annealing temperature of 1075 °C as a function of the Nb and Mo content; The micrographs in the background show representative microstructures of Alloy A (left) and Alloy D (right) without and with precipitates of the Laves phase that has a white color. (**b**) DFT results on lattice misfit between $\gamma$ and $\gamma^{\prime}$ for the alloy ${\textrm{Al}}_{4}{\textrm{Co}}_{26}{\textrm{Cr}}_{19}{\textrm{Fe}}_{18}{\textrm{Ni}}_{27}{\textrm{Ti}}_{6})_{(100-2x)}{\textrm{Nb}}_{x}{\textrm{Mo}}_{x}.$ The micrograph in the background shows a representative $\gamma /\gamma^{\prime}$ microstructure taken from Alloy A (left) with 25 to 28 vol$\%$ of the $\gamma^{\prime}$ phase and Alloy D (right), showing similar $\gamma /\gamma^{\prime}$ microstructure for all alloys. (**c**) Known effects and optimum at. % use of selected elements in CCAs (Al and Ti see Chang et al.^15^, Nb and Mo results of this work), estimated from effects on Ni-based alloys; Al, Ti and Nb highly affect the $\gamma^{\prime}$ phase by increasing volume fraction and antiphase boundary energy. Mo increases solid solution strengthening in the matrix phase. Nb and Mo promote the precipitation of Laves phase at the annealing temperature of 1075 °C. The lattice misfit can be reduced with simultaneous Nb and Mo additions.

The actual alloy design strategy focuses on the controlled introduction of a minor amount of the Laves phase into the microstructure of a reference wrought GCP precipitation-hardened (PH) FeCoCrNi-base alloy. For this purpose, Calphad calculations shown in Fig. 1a have been conducted to estimate present phases and alloy constitutions. Calculations suggest the formation of Laves phase with increasing Mo and Nb content up to a phase fraction of approx. 0.1 with an equivalent content of 2 at.% Nb and Mo at the selected annealing temperature of 1075 °C. Besides the precipitation of Laves phase, DFT calculations shown in Fig. 1b allow for the prediction of lattice misfit between the host fcc alloy matrix and the coherent GCP L1$_2$ precipitates. DFT calculations show that the misfit constantly reduces by alloying with Mo and Nb, suggesting an addition of Nb and Mo also in these calculations. Consequently, from these computational results the content of Nb and Mo to achieve the optimal amount of Laves phase during heat treatment and reduced $\gamma /\gamma^{\prime}$ lattice misfit is predicted to be between 1 and 2 at. % of Nb and Mo, respectively. According to these results, alloys have been produced with a stepwise increase of an equivalent amount of Nb and Mo up to 4 at.%. A broader compositional range has been selected to counterbalance possible deviations between Calphad predictions and the experiment.

The expected influence of the alloying elements on expected material properties is described in Fig. 1c, where the expected alloying effects are derived from the basic knowledge of Ni-based superalloys. As already predicted by the calculations, the literature data supports that Nb and Mo additions to the alloy composition are expected to govern the formation of the TCP Laves phase^26,27^. Additionally, it is expected that Nb further increases the APB energy of the $\gamma^{\prime}$-phase^28,29^ and Mo contributes to the solid solution strengthening (SSS)^17,30^ of the multi-component matrix, further contributing to material strength at elevated temperatures. It is also reported that both elements increase the lattice parameter in the CCA’s matrix^31^, leading to a lower lattice misfit between the $\gamma$ matrix and the $\gamma^{\prime}$-phase, as the latter commonly exhibits a larger lattice parameter in these types of alloys^3,32^. Another essential alloying element is Al, which is considered a key alloying element for the formation of (Ni,Co)_3_(Ti,Al) $\gamma^{\prime}$-phase that gives the alloy’s most significant contribution to an increased strength at elevated temperatures^9^. Additionally, Al, alongside with Cr, contributes to the oxidation resistance of the alloy, forming dense oxide layers at the surface^9^. Ti can substitute Al in the (Ni,Co)_3_(Ti,Al) $\gamma^{\prime}$-phase^33^ and is expected to increase its fraction and solvus-temperature^34^. Additionally, studies report a beneficial increase of the antiphase boundary energy (APB) of the $\gamma^{\prime}$-phase by Ti^28,29,35^. All these effects are expected to further improve the strength of CCAs at elevated temperatures, leading to the produced alloy compositions presented in this work.

### Production and characterization of new CCAs

**Figure 2:**
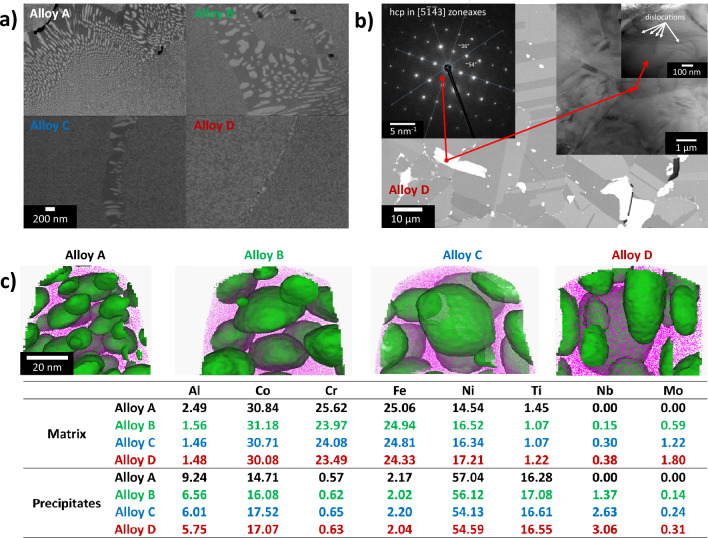
(**a**) SEM backscatter images of the $\gamma /\gamma^{\prime}$ microstructure of alloys A, B, C, and D, where coarser particles at grain boundaries can be found; (**b**) SEM and TEM images of the hexagonal Laves phase in Alloy D; (**c**) APT reconstructions and corresponding matrix and precipitate phase compositions for $\gamma /\gamma^{\prime}$ microstructure of alloys A, B, C and D.

**Figure 3:**
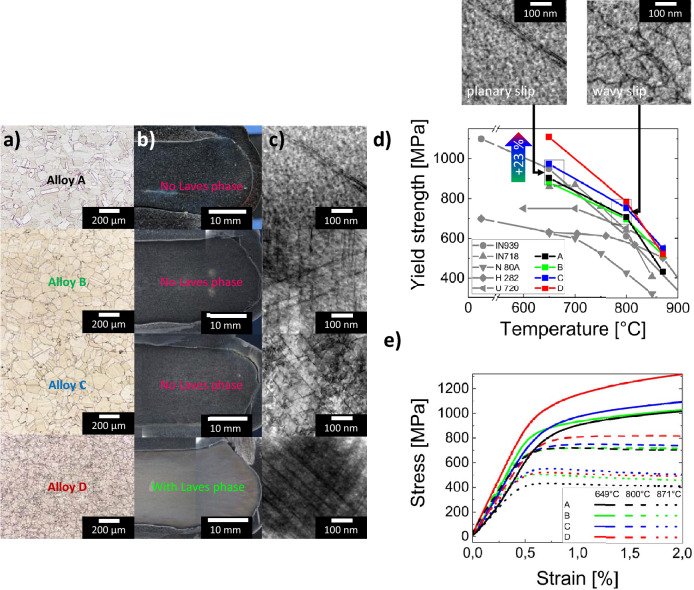
(**a**) Light optical micrograph of the microstructure. (**b**) Macroscopic image of deformed samples directly after thermo-mechanical treatment at 1100 °C, showing successful deformation without fracture of proposed alloys. (**c**) The BF-STEM micrographs of the alloys A-D deformed at 649 °C. (**d**) Temperature dependence of the yield strength compared to Ni-based alloys from literature^40–44^. Due to the lack of compressive yield strengths in literature, the values of Ni superalloys are given in tensile, while the respective CCAs in the focus of this work are in compressive. The insets in (**d**) show BF-STEM images demonstrating the change in dislocation slip with temperature. (**e**) Selected stress-strain curves from compression tests for model alloys A, B, C, and D. Microstructure analysis shows that the laves phase leads to refined grain structure in Alloy D. Deformation mechanism analysis in the TEM show planar dislocation glide at 649 °C as deformation mechanism in all alloys. It seems not to be modified by Nb, Mo, and accompanied laves phases. Yield strength and compression testing results show that alloy D performs better at temperatures below approximately 850 °C than alloys A–C without the Laves phase.

According to the computational design, model alloys (A-D) with increasing Nb and Mo content have been produced in this work via a conventional process, including casting, homogenization, multi-step thermo-mechanical processing, annealing above $\gamma^{\prime}$-solvus and aging^36^. It has been necessary to adapt the homogenization heat treatment for alloy B-D equivalently to homogenize the Mo and Nb content and adjust the amount of Laves phases. Furthermore, it has to be mentioned that alloy A has been subjected to slightly different thermo-mechanical processing compared to the other alloys due to differing initial sample dimensions. See the methods section for more details. The alloys’ chemical compositions are listed in Table 1 and show increasing equivalent amounts of Nb and Mo starting from 0 up to 2 wt.% added to the original alloy composition Al_4_Co_26_Cr_19_Fe_18_Ni_27_Ti_6_. The designed microstructure of all alloys is given in Fig. 2a and c. It shows an intended $\gamma /\gamma^{\prime}$ constitution of the matrix, as well as black nonmetallic inclusions identified as titanium carbides/nitrides caused by processing. The $\gamma^{\prime}$ precipitates in the bulk are found within a size range of 15 to 40 nm in diameter. Their fraction has been determined to be between 25 and 28 vol.%^37^. According to visual inspection in Fig. 2a and c the increasing Nb and Mo content in the studied alloys has been shown to have no significant effect on the morphology and volume fraction but leads to slightly larger $\gamma^{\prime}$ particles in the bulk. In contrast, increased Nb and Mo content appears to result in smaller and more spherical discontinuously precipitated^38,39^ $\gamma^{\prime}$-particles at the grain boundaries. This implies that the discontinuous precipitation^38,39^ of $\gamma^{\prime}$ at the grain boundaries in the designed alloys can be suppressed by alloying with Nb and Mo. A detailed chemical analysis by means of APT reveals that Nb enriches $\gamma^{\prime}$ precipitates, whereas Mo dissolves in the fcc matrix, therefore contributing to further decrease of the lattice misfit (Fig. 1b). Alloy D with the highest amount of added Nb and Mo (Fig. 2b) shows additional nano- to micrometer-sized phases, appearing bright in backscattered SEM imaging. A combination of selected area diffraction (SAD) and EDS measurements directly at these phases evidence the presence of Laves phase with an hexagonal close packed (hcp) crystal structure and rich in Mo and Nb, agreeing with computational results. However, microstructural analysis of alloys A–C with Nb and Mo up to 1 at.$\%$, respectively, has shown no Laves precipitates in these alloys. Consequently, it is proposed that the microstructure of alloy D with approx. 2 at.$\%$ Nb and Mo is substantially different and contains a significant amount of Laves phase precipitates, whereas already in alloy C with approx. 1 at.$\%$ Nb and Mo, the Laves phase precipitation seems to be suppressed at applied processing. The Laves phase shows a size distribution where the larger fraction is in a size range between 10 and 40 $\mu$m with an elongated shape, and the smaller fraction ranges from nanometer to 1-2 $\mu$m and has a spherical shape. The Laves phase is preferentially arranged at grain boundaries as can be seen in Fig. 2b, suggesting a possible Zener pinning effect during processing, allowing for effective grain-refinement as shown in Fig. 3d. Interestingly, dislocations discovered within the Laves phase are found in the BF-STEM images in Fig. 2b. This observation suggests a certain degree of ductility in these phases during deformation, which is unusual for generally brittle TCP phases.

### Properties of new CCAs and effect of the Laves phase

The correlation between the microstructure and mechanical properties has been established in a series of compression tests at various temperatures. Figure 3d and e show that alloy D, containing the Laves phase and approx. 2 at.$\%$ of Nb and Mo, exhibits a 23 $\%$ increase in yield strength at 649 °C compared to the alloys without Nb and Mo addition, achieving a yield strength of 1109 MPa, surpassing all reference Ni-based alloys subjected to tensile mechanical testing. Alloy D also shows, e.g., a 29 $\%$ increase in yield strength compared to Inconel718 (860 MPa). It should be explicitly mentioned that despite the presence of the Laves phase in alloy D, the alloy shows reasonable workability at a typical deformation temperature of 1100 °C, as evidenced in Fig. 3b. Furthermore, compression curves tested at lower temperatures show plasticity beyond 2 $\%$ strain. It should be noted that the material has a higher ductility (than 2 $\%$), as tests were stopped at a strain of 2 $\%$. Despite this fact, the results imply a reasonable ductility of all model alloys. Mechanical testing at 871 °C reveals already lower yield strength variations in the model alloys with a maximum of 549 MPa in alloy C, suggesting promising mechanical strength also at this temperature.

The increased strength at 649 °C of alloy D is a result of a significant contribution of the reduced grain size compared to alloys A-C, which is shown in Fig. 3a. This is primarily a consequence of the presence of Laves phase particles, causing Zener pinning, solute drag effects or possibly particle stimulated nucleation (PSN) during thermo-mechanical processing. SEM investigations shown in Fig. 2a imply a related drop in the grain boundary mobility with Nb and Mo alloying. This is suggested by the identified regions with elongated, coarsened $\gamma^{\prime}$ particles during aging at 750 °C, which are assumed to be a consequence of discontinuous precipitation of $\gamma^{\prime}$ particles, where a high supersaturation of the matrix and mobility of the grain boundaries are essential^38,39^. Their extend decreases constantly with increasing Nb and Mo content, suggesting decreasing grain boundary mobility and increasing $\gamma^{\prime}$ solvus temperature.

Since the type of dislocation motion can affect the strengthening mechanisms and they can modify with Nb and Mo additions, tested alloys were investigated regarding the interaction of dislocations with the coherent GCP $\gamma^{\prime}$-particles, using BF-STEM shown in Fig. 3c and d. Investigations in Fig. 3c show that at a temperature of 649 °C, the chemical variation does not affect on the gliding behavior of dislocations, as the planar dislocation gliding has been found in all alloys. It should be mentioned that dislocations in these images can be distinguished by contrast, whereas the $\gamma^{\prime}$-particles show no contrast to the matrix. At this testing temperature, no twinning is observed, implying that the planar dislocation glide is the only deformation mechanism active in all investigated alloys. This type of mechanism indicates a precipitation hardening mechanism via dislocations cutting through the precipitates, having an antiphase boundary in between^45^. At a testing temperature of 800 °C, a different type of deformation mechanism has been detected. BF-STEM investigations depicted as an insert in Fig. 3d show a so-called wavy dislocation slip in alloy A, circumventing $\gamma^{\prime}$ particles. Again, in these images $\gamma^{\prime}$-particles show no contrast to the matrix, but the appearance of the dislocations suggests this behavior. It is known that the ability of dislocations to circumvent particles generally enhances with increasing temperature, causing a wavy dislocation slip, additionally contributing to temperature-induced softening.

Essential properties of the model alloys A–D are summarized in Table 1. The results show a unique combination of strength at 649 °C, relatively low density, workability, and potential for grain size control, which indicates outstanding potential for application at high temperatures. Alloy D, with the highest amount of Nb and Mo still shows lower density than IN939 or Haynes 282, exhibiting 8.15^46^ and 8.29^47^ g/$\textrm{cm}^{3}$, respectively. All model alloys subjected to thermo-mechanical processing can achieve technologically relevant ASTM grain size numbers^48^, ranging from 4–6 for the alloys A–C to 8–10 for alloy D. Since Laves phases are preferentially arranged at grain boundaries, and the fact that alloys B and C do not show a significant grain refinement, whereas alloy D with Laves phases shows a pronounced refinement, indicates that the presence of the Laves phase in alloy D contributes significantly to the effective grain size control during thermo-mechanical processing. This observation also suggests that a similar effect of the Laves phase is present in $\delta$-processed Ni-based superalloys^49,50^. This grain refinement effect is essential for components exposed to low cycle fatigue at elevated temperatures. The grain sizes achieved in alloys A-C without the Laves phase are technologically relevant for practical applications such as turbine casings.

**Table 1:** Properties of the designed alloys A–D.

Alloy	Composition (at. $\%$)	$\rho$ (g/$\textrm{cm}^{3}$)	$\sigma _y$ (MPa)	ASTM grain size number	Laves phase
A	${\textrm{Al}}_{4}{\textrm{Co}}_{26}{\textrm{Cr}}_{19}{\textrm{Fe}}_{18}\textrm{Ni}_{27}\textrm{Ti}_{6}$	7.95	904	4–6	No
B	$\textrm{Al}_{3}\textrm{Co}_{28}\textrm{Cr}_{18}\textrm{Fe}_{18}\textrm{Ni}_{27}\textrm{Ti}_{5}\textrm{Nb}_{0.5}\textrm{Mo}_{0.5}$	8.00	882	4–6	No
C	$\textrm{Al}_{3}\textrm{Co}_{28}\textrm{Cr}_{17.5}\textrm{Fe}_{17.5}\textrm{Ni}_{27}\textrm{Ti}_{5}\textrm{Nb}_{1}\textrm{Mo}_{1}$	8.01	974	4–6	No
D	$\textrm{Al}_{2.5}\textrm{Co}_{27}\textrm{Cr}_{17.5}\textrm{Fe}_{17.5}\textrm{Ni}_{26}\textrm{Ti}_{5.5}\textrm{Nb}_{2}\textrm{Mo}_{2}$	8.07	1109	8–10	Yes

## Summary and conclusions

In this paper, we demonstrate that new CCAs with $\gamma /\gamma^{\prime}$ microstructure containing Laves phase precipitates have superior mechanical strength at elevated temperatures and show promising results compared to other wrought CCAs as well as wrought Ni-based superalloys not only in terms of high-temperature mechanical strength but also in terms of material weight reduction and grain size control while maintaining workability. These properties are significant in material selection processes for aerospace- and energy-producing applications, making these alloys promising candidates for these applications. In particular, alloy D with the highest Nb and Mo content shows a promising yield strength of 1109 MPa in combination with comparably low density, a fine grain size of 8–10 on the ASTM grain size scale, and reasonable workability during thermo-mechanical processing. Our investigation shows that the desired Laves-phase-reinforced microstructure can be obtained by adding an equivalent amount of 2 at.$\%$ of Nb and Mo to the Al_4_Co_26_Cr_19_Fe_18_Ni_27_Ti_6_ CCA, which can be produced via a casting route with conventional thermo-mechanical processing and heat treatment steps. The alloying elements Nb and Mo, as well as the presence of the Laves phase, retard the grain boundary movement by Zener pinning or solute drag effects during processing and might allow for PSN of recrystallized grains, leading to the refinement of the microstructure to technologically relevant grain sizes. The presence of the Laves phase, as well as chemical variations in the $\gamma /\gamma^{\prime}$ microstructure, do not lead to any change in the deformation mechanism at 649 °C. The TEM investigations in Fig. 2b show that the precipitates of the Laves phase may even exhibit a certain degree of ductility itself, which is not an expected behavior for brittle TCP-type Laves phase precipitates^51^. The successful implementation of the proposed CCA design concept suggests a possible change in the perception of Laves phases as “undesired” in the superalloy design in general. This has already been proposed for ferritic, austenitic steels but is entirely new for CCAs, which show a different composition of the matrix and the Laves phases. Although the effect of Laves phases on the mechanical properties of this material needs to be further investigated, it has been shown that the concept has a high potential for the design of new materials for application at elevated temperatures and can be applied to revisit existing wrought precipitation hardened alloys by including an optimal amount of fine, spherical Laves phase precipitates in their microstructure.

## Methods

### Alloy production

The alloys have been produced by vacuum induction melting. The reference alloy (alloy A) has been cast in a cylindrical cast iron mold with an inner diameter of approximately 120 mm. Subsequent homogenization has been performed at 1150 °C for 12 h. A detailed description of the production and an evaluation of the homogenization process of this alloy is given elsewhere^36^. Thermo-mechanical treatment has been performed on an industrial hydraulic press, whereas the multi-step deformation has been conducted at 1100 °C in radial and axial directions to a final height of 25 mm.

The alloys B, C, and D have been cast in a sand mold with dimensions of $50\times 200\times 400$ mm. Subsequently, the blocks have been homogenized at 1150 °C and 1175 °C for 24 h, respectively. For thermo-mechanical treatment, specimens with 36 mm diameter and 60 mm height were cut from the blocks. They were axially deformed on an industrial hydraulic press in a two-step procedure at 1100 °C, resulting in a final height of the specimens of 25 mm.

Subsequently, all alloys were annealed above $\gamma^{\prime}$ solvus temperature (alloy A at 1050 °C for 30 min and alloys B, C, and D at 1075 °C for 30 min), quenched in water and aged at 750 °C for 50 h.

The chemical composition of the alloys has been determined by inductively coupled plasma-optical emission spectrometry (ICP-OES). For microstructural characterization and mechanical testing, specimens of all alloys in appropriate sizes and geometries have been cut from the aged samples.

### Thermodynamic calculations

The Calphad calculations have been done using the ThermoCalc Software (ThermoCalc 2020a) with the Database TCNi8 for Ni-based alloys. The compositions for the calculations have been derived from a modification of the alloy Al_3.31_Co_27.27_Cr_18.18_Fe_18.18_Ni_27.27_Ti_5.78_ from Chang et al.^38^ in a way that the composition can be given as:

$$\textrm {(Al}_{3.31}\textrm{Co}_{27.27}\textrm{Cr}_{18.18}\textrm{Fe}_{18.18}\textrm{Ni}_{27.27}\textrm{Ti}_{5.78})_{(100-2x)}\textrm{Nb}_{x}\textrm{Mo}_{x}$$

where x has been chosen to be 0, 0.5, 1, 2 and 4.

### Microstructural characterization

Microstructural analysis has been done using the optical light microscope (OLM) OLYMPUS BX51M with the camera system OLYMPUS DP23 and the OLYMPUS Stream Motion 2.5 Software. Specimens for OLM have been metallographically ground and wipe etched with a mixture of 80 ml HCl, 20 ml Glycerine, 5 ml HNO_3_, and 1.5 g CuCl_2_.

SEM characterization has been done on a GeminiSEM ® 450 (Carl Zeiss SMT). The SEM specimens have been analyzed at an ion-sliced cross section using a Hitachi IM4000+ ion slicer.

TEM characterization discs with 3 mm in diameter have been ground and dimpled to a height of 5 $\upmu$m at the thinnest point before final thinning to electron transparency using ion-milling. TEM investigations have been carried out using a JEOL JEM-2200FS at an acceleration voltage of 200 kV.

The APT characterization has been done using rod-shaped specimens cut from the produced model alloys and electropolished to have the final tip geometry of 10–20° shank angle and less than 100 nm in diameter. Measurements have been performed on a LEAP 3000 X HR System from Cameca at 60K and with 20 $\%$ pulse fraction, 200 KHz, and 0.5 $\%$ target evaporation. The IVAS 3.8 software from Cameca has been used for tip reconstruction.

### Mechanical testing

For the compression tests, cylindrical specimens with 8 mm diameter and 12 mm height have been produced from the final state of all alloys and heated inductively in the testing machine. The tests have been performed on an Instron 8803 testing machine (equipped with hard metal stamps) at a strain rate of 2.5*10^-4^ s^-1^ and a maximum strain of 2 $\%$. Precise strain measurement was carried out by means of a laser extensometer. All alloys were tested at 649 °C, 800 °C, and 871 °C, whereas 2 samples were tested for each alloy and testing temperature.

### First principles calculations

The spin-polarized density functional theory calculations have been performed using the EMTO (exact muffin-tin orbitals)^52–54^-LSGF^55^ (locally self-consistent Green function method) method^56^ within the

coherent potential approximation (CPA) for disordered alloys^57,58^. All the self-consistent EMTO-LSGF calculations were performed by using an orbital momentum cutoff of l$_{max}$=3 for the partial waves in the spdf basis with the local interaction zone for electronic multiple scattering processes set to three nearest neighbor shells in the supercell calculations. The total energies were obtained using the GGA-PBE functional^59^. The lattice misfit has been defined using the lattice parameters of $\gamma^{\prime}$ and $\gamma$ as:

1 $$\begin{aligned} \delta&= 2 \times \left[ \frac{a_\gamma \prime - a_\gamma }{a_\gamma \prime + a_\gamma } \right] \end{aligned}$$

The Compositions for the calculations have been derived from a modification of the alloy Al_3.31_Co_27.27_Cr_18.18_Fe_18.18_Ni_27.27_Ti_5.78_ from Chang et al.^38^ in a way that the composition can be given as:

$$\textrm{(Al}_{3.31}\textrm{Co}_{27.27}\textrm{Cr}_{18.18}\textrm{Fe}_{18.18}\textrm{Ni}_{27.27}\textrm{Ti}_{5.78})_{(100-2x)}\textrm{Nb}_{x}\textrm{Mo}_{x}$$

where x has been chosen to be 0, 2, and 4.

## Data Availability

The data that support the plots within this paper and other findings of this study are available from the corresponding author upon reasonable request.
